# Custom CAD/CAM Peek Implants for Complex Orbitocranial Reconstruction: Our Experience with 15 Patients

**DOI:** 10.3390/jcm13030695

**Published:** 2024-01-25

**Authors:** Cristina Cárdenas-Serres, Fernando Almeida-Parra, Anna María Simón-Flors, Patricia de Leyva-Moreno, Álvaro Ranz-Colio, Luis Ley-Urzaiz, Julio Acero-Sanz

**Affiliations:** 1 Department of Oral and Maxillofacial Surgery, Ramón y Cajal University Hospital, IRYCIS, 28034 Madrid, Spainpatricia.leyva@salud.madrid.org (P.d.L.-M.); jacero@salud.madrid.org (J.A.-S.); 2 Department of Oral and Maxillofacial Surgery, Puerta de Hierro University Hospital, 28222 Majadahonda, Spain; 3 Department of Neurosurgery, Ramón y Cajal University Hospital, IRYCIS, 28034 Madrid, Spain

**Keywords:** cranio-orbital complex, bone defects, PEEK, virtual surgical planning, intraoperative navigation, osteotomy, reconstruction, patient-specific implants

## Abstract

Bone defects within the cranio-orbital complex present unique challenges in terms of surgical planning and reconstruction. This article presents a novel approach using PEEK material and advanced surgical technologies to address these challenges. A retrospective analysis of 15 patients who underwent craniofacial reconstruction using patient-specific polyetheretherketone (PEEK) implants between 2016 and 2021 was carried out. Comprehensive preoperative planning was performed, utilizing advanced imaging techniques and specialized software for virtual surgical planning. Patient-specific PEEK PSIs were designed and manufactured based on the preoperative plan. Intraoperative navigation was used to guide the surgical procedure, enabling precise osteotomy and optimal implant placement. This article describes the step-by-step process and the tools utilized in each phase. The etiologies were as follows: meningioma in seven cases, benign lesions in five cases, malignant tumors in two cases, and trauma sequelae in one case. In all cases, 3D-printed PEEK implants were utilized to achieve precise reconstruction. No major complications were described. In one case, an implant replacement was needed with successful outcomes. Our study demonstrates the feasibility and effectiveness of using PEEK patient-specific implants for personalized craniofacial reconstruction. The combination of advanced imaging, virtual planning, and CAD-CAM technology contributes to improved surgical outcomes in terms of oncologic margin control, functional restoration, and aesthetic results.

## 1. Introduction

The aesthetic and functional reconstruction of complex cranio-maxillofacial defects can be challenging, especially involving deformity and tissue loss as a result of trauma, oncologic resection, and craniofacial syndrome. 

The cranio-orbital region serves as a vital support and protective structure for various components, including the eyeball, orbital cavity, brain, internal carotid artery, and cranial nerves. Comprising a pyramid-shaped framework with a quadrangular base, the orbit remains to be a complicated 3D structure which presents a significant challenge for surgical reconstruction and the correction of deformities in this area. Conventional techniques frequently employed for orbital reconstruction involve the use of standard titanium meshes, or polymeric implants, which need pre- or intraoperative bending and contour correction. The precise location of implants and their adaptability to the individual anatomy of the affected structures (in terms of size and shape) are critical factors for the overall success rate in cranio-orbital reconstruction.

With the development of computer-aided design/computer-assisted manufacturing (CAD/CAM), and the advancement of virtual surgical planning and 3D printing, the emergence of patient-specific implants (PSIs) has enabled the precise design, production, and fitting of implants tailored to individual anatomical defects with much more predictable postoperative results [1].

Although reconstruction with autologous tissue (such as bone grafting) was traditionally considered the best option for craniofacial bone repair, the introduction of synthetic materials has allowed further development in the field of reconstructive surgery [2]. In light of the limitations observed with metallic and ceramic biomaterials, there has been a recent introduction of polymers as a viable alternative. Numerous polymers, including polymethyl methacrylate (PMMA), polylactide (PLA), and polyglycolide (PGA), have found wide applications in the field of biomedicine. PMMA is a bone cement that is easy to shape and is relatively inexpensive compared to some other materials, but there is a higher risk of infection associated with this biomaterial, especially in long-term applications. PLA and PGA are biodegradable, which means they gradually break down in the body, become eventually replaced by natural tissue, and carry a lower risk of infection compared to non-biodegradable materials, but may not offer the same immediate stability and strength as other materials. Biodegradable polymers are radiolucent, ensuring that they do not interfere with X-rays or CT scans for accurate postoperative assessment. These polymer materials can be used for various reconstructive procedures, including orbital floor and zygomatic arch reconstruction, and they can be combined with other materials when necessary. 

Among the various alloplastic materials, polyether ether ketone (PEEK) has emerged as an appealing choice for PSIs. PEEK is a polyaromatic semi-crystalline thermoplastic polymer that contains ether and ketone linkages. In recent years, it has increased in popularity due to its bone-like strength and elasticity, and other characteristics such as lower thermal conductivity and lower infection rates compared to other biomaterials [3]. PEEK is a lightweight material, making it suitable for facial bone reconstruction, and is radiolucent, allowing for better postoperative imaging. Surgeons should also consider that PEEK is less malleable than metals, which can make it more challenging to shape during the procedure, and can be relatively costly compared to other materials.

The utilization of advanced imaging techniques for the design of PSIs and preoperative planning has become standard practice in complex craniofacial procedures. While the use of imaging data for device design is well established, in our study, we aimed to highlight the specific workflow and considerations related to zygomatic–orbital complex reconstruction, with an emphasis on addressing the complexities and challenges of this procedure. There is currently a lack of systematic reporting on clinical studies regarding the implementation of patient-specific individual PEEK implants for cranio-orbital-zygomatic reconstruction. Hence, the objective of this article is to evaluate our approach for addressing bone defects within the orbito-zygomatic complex using PEEK and share our firsthand experience in employing virtual surgical planning and intraoperative navigation to conduct precise osteotomy and achieve accurate reconstruction through the utilization of custom-made prefabricated PEEK PSIs.

## 2. Materials and Methods

### 2.1. Patient Data

**Figure 1:**
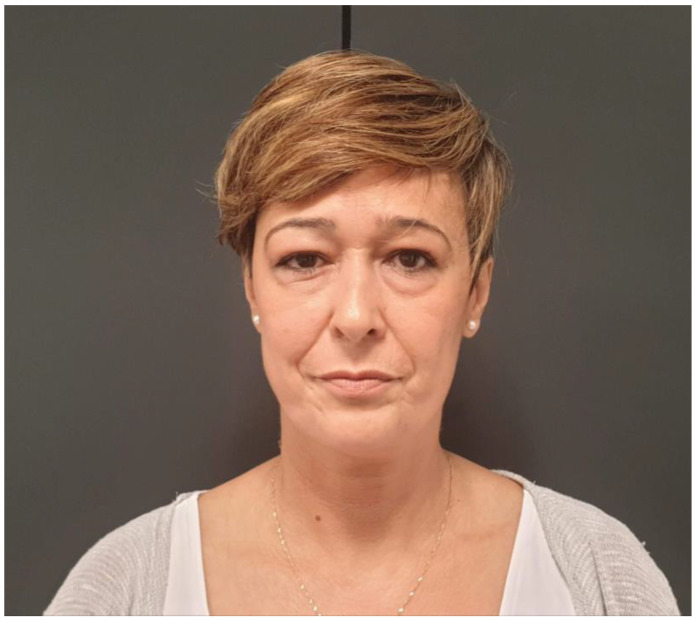
Preoperative imaging of Patient 5 reveals noticeable right ocular proptosis.

This study included 15 patients who underwent craniofacial reconstruction using PEEK PSIs in our department between 2016 and 2021. All the patients enrolled presented with either a benign or malignant lesion that needed complex cranioorbital resection or the subsequent reconstruction of the resulting defect. The variables analyzed were the sex, age, medical history, etiology, size, and location of the defect. The type of reconstruction performed, i.e., primary or secondary, and any postoperative complications were also recorded. Preoperative demographic data, as well as clinical and radiological findings, are presented. After obtaining informed consent, preoperative clinical images were taken of all patients with the intention of attaining an outcome closely resembling their preoperative state (Figure 1). Data were obtained retrospectively from hospital, clinical, and surgical records. The mean follow-up was 2.5 years, and ranged between 2 and 7 years.

### 2.2. Preoperative Study and Virtual Surgical Planning

**Figure 2:**
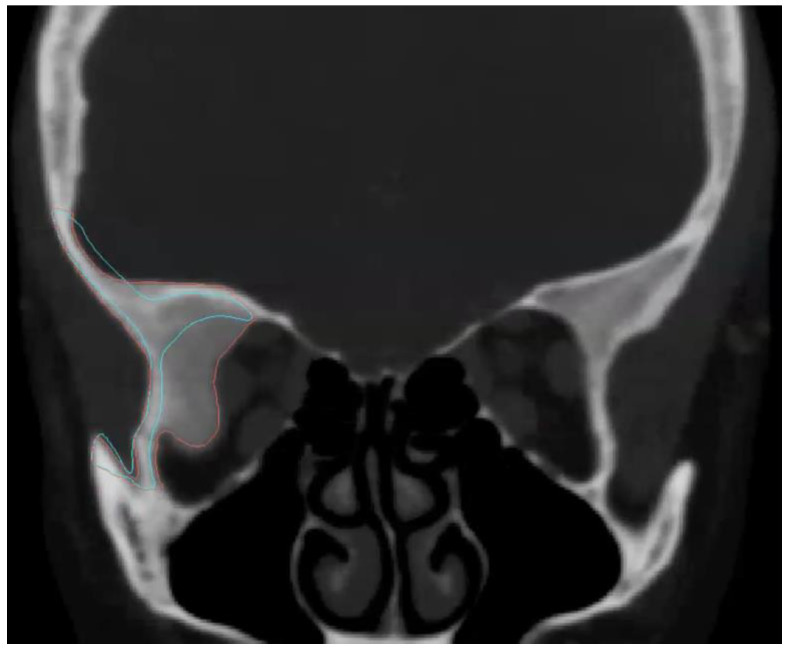
The preoperative CT scan of Patient 5 indicates a lesion consistent with meningioma in the fronto-orbital region (highlighted in red). The virtual design of the PEEK PSI is represented in blue.

Multislice computed tomography (slices < 1 mm) was performed in all patients as part of a preoperative study (Figure 2). A 3D study of the patient was obtained using the Digital Imaging and Communications in Medicine (DICOM) viewer and was imported to computer software (Brainlab I-plan 3.0^®^, Munich, Germany) where virtual surgery was performed. The PSI design process was a collaborative effort, closely involving both the surgical team and biomedical engineers, with the primary objective of achieving precise and optimal outcomes. Preoperative CT data in DICOM format were used, ensuring that the files were uncompressed to maintain the highest possible quality and accuracy of anatomical information. These images served as the foundation for our PSI design process. The interdisciplinary collaboration between biomedical engineers and the surgical team allowed us to leverage the expertise of both parties. Surgeons provided critical insights into the anatomical requirements, the specifics of the craniofacial defects, and the desired placement of the implant to achieve optimal functional and aesthetic results. This collaboration ensured that the implants were tailored to each patient’s unique anatomy and needs.

The biomedical engineers utilized the preoperative CT data to design the PSI, considering factors such as implant size, shape, and optimal positioning within the orbit. In one-step reconstructions, a combination of multiplanar two-dimensional (2D) slices and three-dimensional (3D) volume-rendering models was utilized to meticulously delineate the lesion and establish surgical bone resection margins with precision before the PSI was designed. For secondary delayed reconstructions, software was employed to transform and manipulate the CT data, enabling the generation of an anatomically appropriate implant. In instances of unilateral cases, whenever feasible, a mirroring technique was applied.

Once the design was finalized and approved by the surgical team, the PSI was manufactured through a milling process from radio-opaque PEEK blocks. The use of this material, which resembles bone in terms of density, provided an additional advantage in terms of the radiographic monitoring and assessment of implant positioning.

The collaborative design and manufacturing process ensured that the PSI was tailored to each patient’s specific needs, taking into account the intricacies of their craniofacial defects. This approach not only improved the accuracy of implant placement but also enhanced the overall outcomes in terms of aesthetics, functionality, and postoperative quality control.

**Figure 3:**
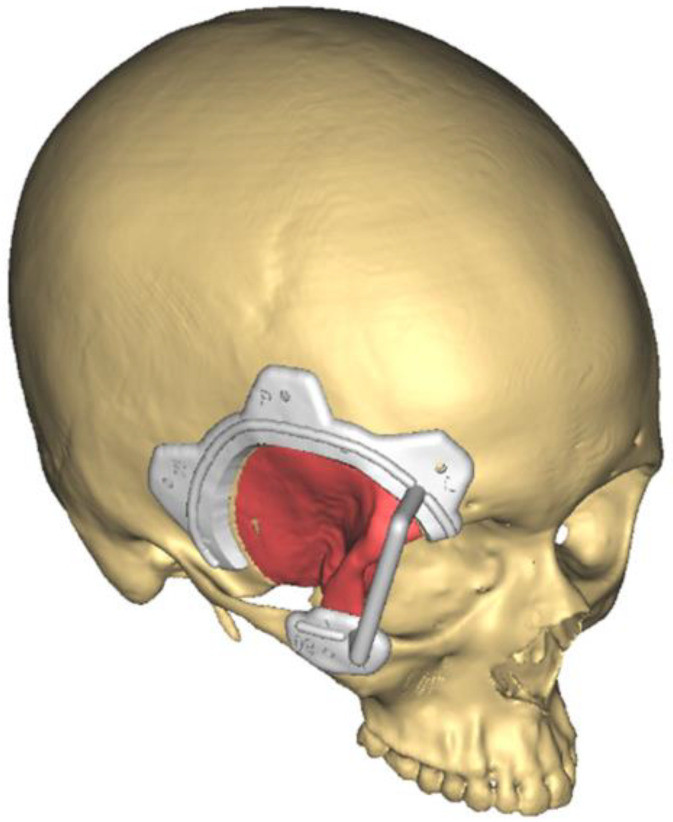
The virtual surgical planning for Patient 5 involved a fronto-temporal craniectomy (red), assisted by a designed and manufactured cutting guide (white).

Virtual planning was transferred into the surgical field through navigation (Brainlab I-plan) or surgical guides, performing the planned resection and the immediate insetting of the custom-made implant. Figure 3 shows the virtual surgery planned for Patient 5, focusing on meningioma excision. The planned procedure involved a fronto-orbital craniectomy, and to facilitate this, a surgical cutting guide was meticulously designed through collaboration between the surgical team and biomedical engineers. This cutting guide was instrumental in ensuring precision during the craniectomy, aligning with the patient’s unique anatomy and the requirements of the surgical plan.

Using intraoperative navigation, a non-invasive registration process for correlating anatomical references to digitalized CT was performed. Skin markers at various points of the face or surface matching were alternatively used, and the register was performed preoperatively.

### 2.3. Surgical Procedure

**Figure 4:**
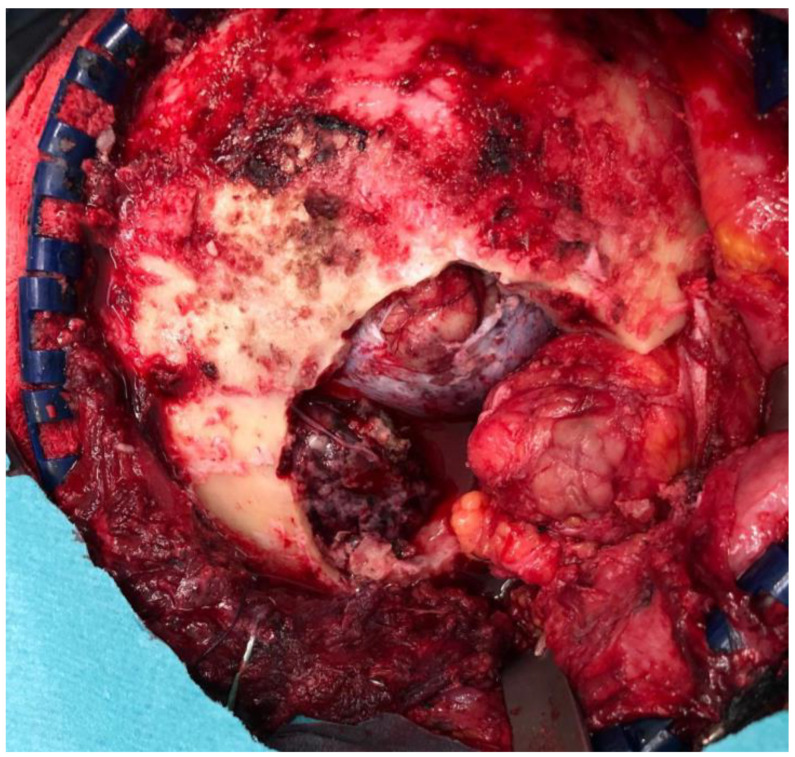
In the case of Patient 5, a right hemicoronal incision was meticulously carried out to provide optimal exposure for the subsequent fronto-temporal craniectomy, and surgical field post-tumor resection can also be observed.

**Figure 5:**
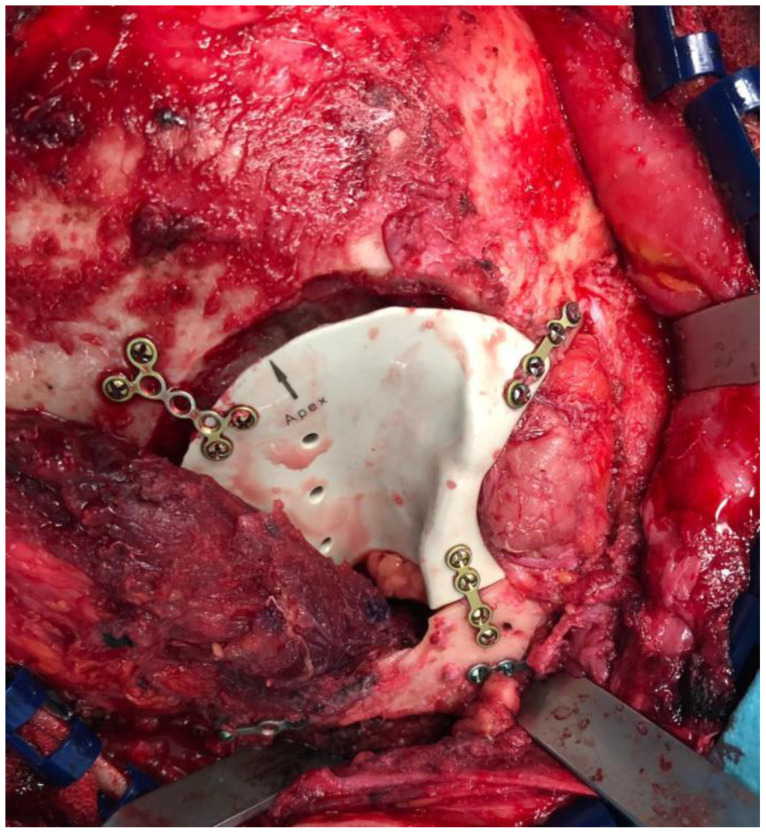
After the successful resection of the tumor in Patient 5, the pre-fabricated PEEK implant was placed as planned.

All the procedures were performed under general anesthesia. An extraorbital–transcranial approach was used for all the patients. Bicoronal, hemicoronal, and intraoral incision types were used to expose the orbital rim and zygoma region. According to the location of the lesion, different approaches were performed, classified into four groups: the anterior approach (fronto-orbital craniotomy), the lateral approach (temporo-orbito-zygomatic), the anterolateral approach (fronto-temporal and fronto-orbito-zygomatic), and combined approaches (orbito-malar). Neither the endoscopic approach nor the transfacial approach was needed. For Patient 5, a hemicoronal incision was executed to facilitate the fronto-temporal craniectomy. Figure 4 shows the surgical field after tumor resection, revealing the defects. The entire process was guided by surgical guides designed beforehand, ensuring precision and adherence to the planned resection margins. Following the tumor resection, the previously manufactured PEEK implant was placed into the defect and securely fixed to the adjacent bone with titanium miniplates. In this instance, the noticeable gap between the implant and the underlying bone surface can be attributed to the neurosurgeon executing a craniotomy that was wider than initially planned, driven by technical considerations (Figure 5). Intraoperative complications were recorded retrospectively.

Intraoperative or postoperative cranial CT examination was performed in all cases to check the planned tumor resection and the correct PEEK PSI position. The expected resection and planned reconstruction were compared with the radiological results. 

All the resected lesions were sent for the histopathological study and the results were collected.

### 2.4. Follow-Up

**Figure 6:**
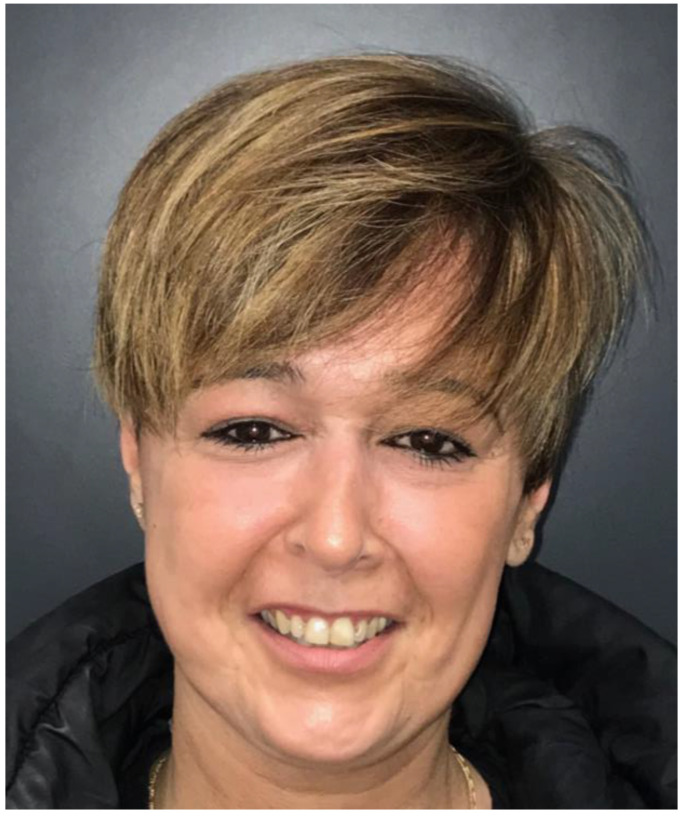
Patient 5 after 6-month follow-up.

All patients were submitted to regular follow-up examinations in the first month after the procedure and subsequently every 6 months to evaluate potential recurrence, functionality, and aesthetic outcomes through clinical assessments (Figure 6). A CT scan was performed during the 12-month follow-up to assess tumor recurrence and the PEEK PSI position. Any postoperative complications, including ocular mobility restrictions, diplopia, allergic reactions, etc., were also recorded.

The analyzed data are presented descriptively, with a review of the scientific literature on the topic.

## 3. Results

The reconstruction of cranio-orbital defects using virtual surgical planning and custom-made PEEK PSI was performed on 15 patients (12 female and 3 male), with an average age of 46.13 years (ranging between 18 and 66 years). The genders, ages, preoperative clinical findings, and pathological diagnosis are shown in Table 1.

Meningioma was the most frequent etiology (seven cases—46%), followed by benign bone lesions (three cases—20%), other benign tumors (two cases—13.33%), malignant tumors (two cases—13.33%), and trauma sequelae (one case—6.66%). The extension of the defect measured in the preoperative CT scan after virtual surgical planning ranged from 10.01 cm^3^ to 256.5 cm^3^ (mean surface 61.37 cm^3^).

An extraorbital–transcranial approach was selected in all patients, using hemicoronal incision (10 cases—66.6%), coronal incision (3 cases—20%), and combined hemicoronal-intraoral approach (2 cases—13.33%). In 12 patients, the lesion resection was performed with immediate reconstruction using PEEK PSIs, and in the other 3 patients, a delayed reconstruction was performed. The mean operative time was 369 min. Wound healing was observed in all patients with no complications. The median hospital stay of the patients included in this study was 4.9 days (range: 2–11 days). 

The aesthetic outcomes in our study were good, characterized by the absence of cranial convexities and orbital rim asymmetries. During the first three months, temporal asymmetry could be observed in most of the patients due to postoperative edema, but it spontaneously resolved during follow-up. In a single case, our study encountered less favorable outcomes, primarily stemming from an inadequate relationship between the soft tissue cover and the volume of the implant. Lipo-filling was performed one year after primary reconstruction was performed with successful outcomes.

Only one intraoperative complication was recorded: one of the patients (6.66%) showed malposition in the implant due to failed navigation relating to the setting of the stereotactic system. A second surgical procedure was needed to replace the implant in the correct position with favorable outcomes. In the two cases, including the one discussed in the article, the implant’s contour deviated from the planned surgical defect as a result of the necessity for an expanded craniotomy performed by the neurosurgeon. Postoperative complications were also analyzed. During the first 6-month postoperative, mild complications were registered, mostly edema (10 cases—66.6%), ecchymosis (8 cases—53.3%), and diplopia (3 cases—20%), with complete resolution and without the need of reintervention. PEEK PSI infection only occurred in one patient (6.66%), presenting wound dehiscence and exposure of the osteosynthesis material used for the fixation of PEEK PSIs in the superior orbital rim. The osteosynthesis material was removed in a second surgical procedure conserving PEEK PSIs with no further complications. There was no surgical mortality. No recurrence of the lesion was observed during the follow-up. Postoperative complications are shown in Table 2.

Patients reported high levels of satisfaction with the aesthetic results achieved through the utilization of patient-specific PEEK implants. They expressed relief and contentment with the improved appearance of their craniofacial region. Beyond aesthetic improvements, patients also noted that the restoration of their facial appearance positively influenced their overall quality of life. They reported feeling more confident and self-assured in social and professional settings.

Long-term follow-up revealed that the aesthetic benefits of patient-specific PEEK implants remained stable over time. This element of sustainability added to the overall satisfaction, as patients could enjoy lasting improvements.

## 4. Discussion

Trauma, chronic infections, and malformation syndromes are the main causes of defects in the cranio-orbital region, with benign and malignant tumors representing the most frequent type of etiology in our series. Orbito-cranial neoplasms can be of primary origin, and secondary tumors and metastasis tumors, being primary orbit lesions, are the most frequently described in the literature [4]. In our case series, the majority of cases showed the secondary origin of tumors arising from surrounding anatomical regions. This can be attributed to this study’s inclusion of only large tumors that required an aggressive approach and extensive resection, including craniofacial bone osteotomies.

Meningioma is the most frequently observed lesion in our series. It is the most common primary tumor of the central nervous system (CNS) and constitutes up to 55% of non-malignant primary CNS tumors [5]. Despite being a benign lesion, meningiomas can lead to certain morbidity, particularly those with an aggressive growth pattern.

To thoroughly assess each case, imaging tests such as computed tomography (CT) and magnetic resonance imaging (MRI) with axial, coronal, and sagittal plane reconstruction should be performed. Obtaining 3D images will provide us with a deeper understanding of the anatomical relationships between the lesion to be resected and other structures, such as eye globes, cavernous sinus lesions, or internal carotid arteries [1,6].

### 4.1. Surgical Approach

When selecting the optimal surgical approach for adequate resection and reconstruction, factors such as the anatomical location, size, and type of the tumor must be carefully considered. Numerous surgical approaches have been well documented in the literature [1]. For benign or smaller tumors located in the midline of the anterior skull base, a trans-nasal endoscopic approach can be a viable option. However, for major benign lesions or malignant tumors, as presented in our study, alternative surgical approaches should be chosen, including the coronal approach, the lateral approach, the anterolateral approach, or a combination of these approaches [4,7].

In order to achieve wide surgical exposure, one or more osteotomies may be necessary. For instance, in the coronal approach, frontal craniotomies are typically performed, minimizing neural tissue retraction. When dealing with tumors superolateral, superomedial, or inferolateral to the optic nerve, a lateral approach with temporo-orbital-zygomatic osteotomy is often preferred [8]. Additionally, if required, transfacial or transmandibular approaches can also be considered.

### 4.2. Surgery Virtual Planning: CAD CAM Technology

Advances in CAD/CAM technology have led to an evolution in cases involving the reconstruction of cranio-maxillofacial defects. By utilizing CAD/CAM technology, surgeons can establish accurate pre-operative plans, conduct virtual ablations, and plan osteotomy and reconstruction procedures. This advancement has allowed for improved aesthetics and functionality through more precise surgical procedures and reduced operation times [9,10].

At our institution, three-dimensional facial analysis and virtual surgical planning were incorporated into all of our cases involving orbito-craniomaxillofacial reconstruction and ablation over the past few years. CT scan multislice images were transformed into three-dimensional (3D) digital imaging and were then converted into a standard triangle language (STL) format using CAD technology. Through the 3D study, we could accurately delineate the lesion to be resected and establish safe oncologic margins prior to surgical intervention [11,12,13]. Additionally, it was feasible to conduct preoperative virtual surgery, incorporating the surgical approach, resection osteotomies, and the manufacture of computer-generated cutting guides based on the planned procedure. The collaborative process between biomedical engineers and the surgical team was integral to ensuring precise implant design and optimal patient outcomes. By transferring virtual surgery to the operating room, either through intraoperative navigation or the utilization of cutting guides, we could achieve the desired outcomes, as shown during the planning phase. 

Over the past decade, there has been a notable increase in the use of intraoperative navigation applications in head and neck surgery. This trend can be attributed to the intricate anatomy of this region and the imperative for precise outcomes. These stereotaxy systems enable the accurate localization of anatomical landmarks or implants with a margin of error ranging from less than 1 to 2 mm [14]. This heightened surgical precision enhances safety by allowing us to effectively manage the anatomical relationships between the tumor and vital structures (like the cavernous sinus or the internal carotid artery) [15]. One potential drawback of intraoperative navigation is that if the stereotactic system becomes displaced during the surgical procedure, it can lead to an error during the resection or placement of the custom implant, as occurred in one of our cases.

Although further prospective studies with larger patient cohorts are necessary, the use of intraoperative navigation appears to contribute to the improved control of surgical margins, particularly in tumors situated within complex anatomical regions, like the cranio-orbital region or the skull base [13,16].

Surgical guides can be designed and manufactured according to our virtual surgery. By combining the use of cutting guides and intraoperative navigation, it is possible to achieve safer resection margins, enhance intraoperative precision, and reduce overall operative times [1,9,11,15].

### 4.3. Reconstruction with Patient-Customized Implants (PSIs)

Other advantages of CAD/CAM technology include enhanced accuracy in achieving aesthetic results and the ability to restore large and geometrically complex anatomical defects through the design and creation of patient-specific implants. The design process of patient-specific implants (PSIs) in our center involves a series of essential steps. The process starts with an in-depth preoperative assessment of the patient’s cranio-maxillofacial defects, typically utilizing various diagnostic modalities, such as CT scans and three-dimensional (3D) imaging. These images provide precise information about the extent and shape of the defect and any surrounding structures that must be considered [8,17,18]. Collaboration among the surgical team, including craniofacial and maxillofacial surgeons, as well as biomedical engineers, is essential. The surgical team’s expertise guides the implant’s functional and anatomical requirements, while the engineers contribute their knowledge of materials and design techniques. The implant design process involves sculpting a prosthetic piece that precisely matches the patient’s unique defect. The implant should not only be anatomically accurate, but also capable of restoring lost functionality, such as providing structural support or maintaining occlusion in the maxillofacial region.

The implant’s design incorporates safe margins, ensuring that it extends beyond the edges of the defect to guarantee complete coverage. This margin is typically a few millimeters and aids in preventing any potential complications or adjacent tissue exposure. The design should also account for any surgical hardware, such as screw holes or attachment points. These facilitate the fixation of the implant during surgery to ensure stability. The designed implant should undergo rigorous validation to confirm its fit and accuracy. This may involve 3D-printing a prototype of the implant to ensure that it aligns precisely with the patient’s defect. Once the design is validated and approved, the final implant is manufactured. The design data are sent to a specialized manufacturing facility, where the implant is fabricated with precision using computer-aided machining techniques. Quality control procedures are applied to the manufactured implant to ensure it meets the required specifications. This may involve rigorous testing to guarantee its structural integrity and biocompatibility.

A primary constraint associated with preoperative customized implants is the potential need to deviate from the initially planned approach during surgery. This deviation may arise due to various factors, including the surgeon’s technical considerations, the necessity for a broader resection prompted by intraoperative requirements, or challenges encountered during osteotomy. Surgeons should be mindful of these possibilities, aiming to execute the operation as closely as possible to the initial plan. Nevertheless, the paramount objectives remain, ensuring appropriate oncologic resection margins and prioritizing patient safety.

The complex three-dimensional anatomy of the orbito-cranial region contributes to technical challenges in surgical reconstruction. The gold standard for the bone reconstruction of this region has been conventionally autologous bone due to its biocompatibility and strength, aligning well with native bone characteristics. However, limitations in shaping the graft, potential donor site complications, the lack of predictability, and the time-consuming harvesting process pose difficulties in the reconstruction of defects, especially those that are large or irregular in the orbito-maxillofacial region [1,19]. As a result, alloplastic materials are currently preferred due to their absence of donor site morbidity, intraoperative adaptability, and the advantage of prefabrication through computer design that allows better morphological results to be achieved [8,20,21].

For this purpose, a wide range of materials, including titanium, hydroxyapatite, poly-DL-lactic acid (PDLLA), and polyether-ether-ketone (PEEK), have been used [6,20]. Among these options, PEEK is preferred by the authors for cranio-facial bone replacement. This biomaterial was first developed in 1978 and has been used for surgical reconstruction since 1998 [22]. Since then, PEEK has been extensively utilized in various applications due to its similar strength and weight to human bone, as well as its low infection and allergic reaction rates. Moreover, PEEK is radiolucent and does not generate artifacts in imaging tests, enabling effective post-surgical oncologic monitoring. PEEK prostheses can be precisely molded to match the size and shape of the defect to be covered [8,23]. When compared to other biomaterials such as titanium, both of them exhibit strength, rigidity, biocompatibility, and non-allergenic properties. They can be easily sterilized through heat or ionizing radiation and can be individually manufactured to fit each patient’s needs [24,25]. However, PEEK offers several advantages over titanium. It closely resembles bone in terms of elasticity and density, reducing shielding. PEEK implants can be easily adjusted during surgery, unlike prefabricated titanium implants. PEEK allows increased thickness to restore bone volume and minimize dead space. Unlike titanium, PEEK does not osseo-integrate with bone, requiring fixation, normally using titanium screws to maintain stability and prevent bulging [17,26,27]. PEEK PSIs demonstrated excellent biocompatibility in our series. During the close follow-up of reconstructed patients, no signs of rejection were observed.

According to the related literature, it is essential to acknowledge that PEEK, while offering numerous advantages for cranio-facial bone replacement, is not without its disadvantages. Notably, PEEK can be a relatively costly material which may impact its accessibility and utility in certain healthcare settings. Additionally, one of the notable drawbacks of PEEK is its limited osteointegration potential, which increases the risk of dislodgment and infection, posing challenges in long-term stability [17,26,27]. This is in contrast to materials like titanium which exhibit more favorable osteointegration characteristics [25].

Another concern highlighted in the literature is the comparatively higher infection rate associated with PEEK when compared to titanium implants. This raises concerns about patient safety and long-term outcomes. Furthermore, there have been previous reports of foreign body reactions to PEEK implants, although the incidence remains relatively rare. Such reactions, when they do occur, can complicate the recovery and necessitate additional interventions. In our series, it is noteworthy that only one patient, constituting 6.66% of the cases, experienced an infection related to the PEEK PSI. This isolated incidence of infection is relatively low in the context of our study, and while it represents a potential drawback of using PEEK implants, it is important to consider the specific circumstances and contributing factors that may have led to this outcome.

Moreover, the structural properties of PEEK, including its thickness and lack of porosity, can present challenges in certain clinical scenarios [25]. Specifically, its non-porous nature may impede fluid drainage when required, potentially leading to complications during the healing process. 

Despite these limitations, it is important to recognize that the choice of implant material should be based on a thorough evaluation of the specific patient’s needs, the nature of the procedure, and the surgeon’s expertise. PEEK, with its distinct set of advantages and disadvantages, represents a valuable option in cranio-maxillofacial surgery. Careful consideration of these factors is crucial in achieving the best possible outcomes for patients while minimizing associated risks.

### 4.4. Intraoperative Imaging

Upon the completion of the reconstruction, whether assisted by a navigation system or not, it is essential to submit the surgical outcome to three-dimensional validation for quality control purposes. Ideally, this validation should take place intraoperatively immediately after the reconstruction is finished in order to identify and correct an implant mispositioning, as this happened in one of our cases [1,28].

Intraoperative CT offers a clear advantage for the control of orbito-cranial reconstruction over other imaging modalities, including MRI, due to its high resolution and the adequate visualization of the thin bony structures of the orbit and the implanted materials [29,30]. However, intraoperative CT has some drawbacks, including relatively high radiation doses and high procurement costs [31].

### 4.5. Esthetic, Socio-Psychological, and Functional Results

The socio-psychological adaptation of patients to surgery and the changes in their appearance are critical aspects of the overall well-being and recovery process. Patients undergoing cranio-orbitofacial surgery often face significant changes in their facial appearance, which can have profound effects on their psychological and emotional state. Understanding how patients adapt to these changes, their anxiety levels, and pain tolerance is crucial for providing comprehensive care.

Many patients may experience feelings of distress, sadness, or a sense of identity loss. They may fear societal judgment or stigmatization and face altered self-esteem due to their altered appearance. The fear of the unknown, concerns about surgical outcomes, and the anticipation of potential pain or discomfort can contribute to heightened anxiety levels. During our study, we provide psychological support and counseling in order to help patients adapt to their altered appearance.

Patients’ pain tolerance can vary significantly. Effective postoperative pain management is a key component in helping patients adapt to their new appearance. The surgeons and healthcare team at our center collaborate to develop pain management strategies tailored to each patient. This may include medications, physical therapy, and psychological interventions to improve pain tolerance and enhance recovery.

The overall satisfaction of our patients following craniofacial reconstruction with PEEK PSIs was a fundamental aspect of our study. Unlike conventional reconstruction techniques, which often lead to noticeable aesthetic changes, patient-specific PEEK implants allowed for subtler and more natural enhancements. Patients included in our study reported minimal psychological distress or discomfort associated with their postoperative appearance. Long-term follow-up revealed that the aesthetic benefits of patient-specific PEEK implants remained stable over time. This element of sustainability added to the overall satisfaction, as patients could enjoy lasting improvements.

## 5. Conclusions

The use of CAD/CAM technology has significantly enhanced the evaluation and surgical planning of craniofacial complex tumor resections, enabling the precise design of resection and reconstruction procedures. Through the introduction of cutting-guides and intraoperative navigation, virtual surgical plans can be seamlessly translated to the operating room, facilitating improved control over surgical margins and enhanced proximity to vital structures. The use of customized PEEK implants, along with navigation-assisted techniques, allows for the immediate reconstruction of large craniofacial defects while minimizing the occurrence of major complications and avoiding donor site morbidity. Patients’ emotional responses, anxiety levels, and pain tolerance must be carefully assessed and managed to ensure a successful recovery.

## Figures and Tables

**Table 1:** Patient data, etiologies, locations, and surgical approaches.

N	Age	Gender	Etiology	Clinical Findings	Location	Surgical Approach	Reconstruction
1	18	F	Parry–Romberg syndrome	Asymmetry	Fronto-orbitary	Anterior (FO) ^1^	Immediate
2	66	F	Fibrous dysplasia	Asymmetry	Fronto-Orbitary	Anterior (FO) ^1^	Immediate
3	46	M	Squamous cell carcinoma	Surgical defect; pain	Ethmoid bone	Anterior (F) ^2^	Delayed
4	25	F	Treacher Collins syndrome	Asymmetry	Fronto-orbitomalar	Combined (FOM) ^3^. IO ^4^	Delayed
5	46	F	Meningioma	Ocular proptosis	Greater sphenoid wing	Lateral (TZ) ^5^	Immediate
6	59	F	Meningioma	Hypoacusia; pain	Temporal fossa	Lateral (TZ) ^5^	Immediate
7	47	M	Pleomorphic adenoma	Ptosis; pain	Lacrimal gland	Antero lateral (FT) ^6^	Immediate
8	61	F	Meningioma	Ocular proptosis	Fronto-orbitary	Antero lateral (FOT) ^7^	Immediate
9	50	F	Meningioma	Ocular proptosis	Fronto-orbitary	Lateral (TOZ) ^8^	Immediate
10	34	F	Meningioma	Ocular proptosis; loss of visual acuity	Greater sphenoid wing	Combined (OTM) ^9^	Immediate
11	52	F	Hemangioma	Asymmetry	Orbitomalar	Combined (TZM) ^10^	Immediate
12	53	F	Meningioma	Ocular proptosis	Greater sphenoid wing	Antero lateral (FOZ) ^11^	Immediate
13	63	F	Meningioma	Asymmetry	Temporal fossa	Lateral (TZ) ^5^	Immediate
14	36	F	Liposarcoma	Ocular proptosis; [pain]	Temporo-orbital	Lateral (TOZ) ^8^	Immediate
15	36	M	Trauma sequelae	Asymmetry	Orbitomalar	Combined (TZM) ^10^. IO ^4^	Delayed

^1^ FO: fronto-orbitary. ^2^ F: frontal. ^3^ FOM: fronto-orbito-malar. ^4^ IO: intraoral. ^5^ TZ: temporo-zygomatic. ^6^ FT: fronto-temporal. ^7^ FOT: fronto-orbito-temporal. ^8^ TOZ: temporo-orbito-zygomatic. ^9^ OTM: orbito-temporo-malar. ^10^ TZM: temporo-zygoma-malar. ^11^ FOZ: fronto-orbito-zygomatic.

**Table 2:** Postoperative complications during the follow-up.

Postoperative Complications	N	Patient Number	%
Edema	10	1, 2, 3, 5, 7, 9, 11, 12, 14, 15	66.6%
Ecchymosis	8	1, 2, 4, 5, 7, 8, 9, 11	53.3%
Diplopia	3	1, 8, 9	20%
Reintervention needed			
1. PSI misposition	1	10	6.66%
2. PSI infection	1	3	6.66%
3. Refinements	2	9	13.33%
Life-threatening complications	0		0%

## Data Availability

Data are contained within the article.
